# Understanding the disease burden and unmet needs among patients with cutaneous lupus erythematosus: A qualitative study^☆^^☆☆^

**DOI:** 10.1016/j.ijwd.2018.01.002

**Published:** 2018-04-13

**Authors:** M.E. Ogunsanya, C.M. Brown, D. Lin, F. Imarhia, C. Maxey, B.F. Chong

**Affiliations:** a College of Pharmacy, University of Oklahoma Health Sciences Center, Oklahoma City, Oklahoma; b College of Pharmacy, The University of Texas at Austin, Texas; c Department of Dermatology, University of Texas Southwestern Medical Center, Dallas, Texas; d College of Natural Sciences, The University of Texas at Austin, Austin, Texas

**Keywords:** cutaneous lupus erythematosus, outcomes, unmet needs, qualitative, focus group, burden, quality of life

## Abstract

**Background:**

Cutaneous lupus erythematosus (CLE) is a rare dermatologic autoimmune disease marked by photosensitive lesions that can vary in appearance depending on the subtype. The extent to which CLE affects a patient’s quality of life (QoL) has not been fully characterized. Focus groups were conducted to explore patients’ perspectives of how CLE has affected their lives and to understand the unmet needs in regards to CLE treatment and care.

**Methods:**

This qualitative study involved three focus groups with a total of 19 patients with CLE. A moderator guide containing open-ended questions was used to assess how CLE affects overall QoL. The focus groups were audio-recorded with notetaking. Data were content-analyzed to identify emergent themes.

**Results:**

Four themes emerged as important to patients with CLE: disease sequelae, social interactions, functioning, and unmet needs. Most patients reported decreased QoL due to signs and symptoms such as dyspigmentation and scarring. Having CLE negatively affected patients’ mental health and personal relationships and led to negative coping strategies, such as recreational drug use. Issues related to body image were also elicited by patients. Patients cited unmet needs including lack of treatments to improve chronic skin lesions of CLE and inadequate patient education on living with CLE.

**Conclusions:**

Providers can look for signs of QoL impairment in patients with CLE by asking questions related to body image, mental health, social isolation, and coping mechanisms. Future QoL measures can include the effect of CLE-specific attributes such as scarring and dyspigmentation to empower patients’ voices in determining therapeutic efficacy in future clinical trials. Findings from our study have added a new understanding of daily experiences that were elicited directly from patients with CLE.

## Introduction

Cutaneous lupus erythematosus (CLE) is a chronic and rare dermatologic autoimmune disease that affects more than 200,000 adults in the United States (Durosaro et al., 2009, Jarukitsopa et al., 2015). CLE disproportionately affects more women than men (Klein et al., 2011, Vasquez et al., 2013). The prevalence of CLE ranges from 70.4 (Jarukitsopa et al., 2015) to 73.2 (Durosaro et al., 2009) per 100,000 with an incidence rate that ranges from 4.2 (Jarukitsopa et al., 2015) to 4.3 (Durosaro et al., 2009) per 100,000.

Treatment options available to patients with CLE are limited due to toxicities (Klein et al., 2011). Common symptoms experienced by patients with CLE include photosensitivity, itching, burning, and alopecia (Eastham and Vleugels, 2014, Hansen and Dahle, 2012). These manifestations vary on the degree of severity and persistence depending on the stage and progression of the disease. Painful skin lesions and disfiguration are considerable sources of morbidity among patients with CLE (Kole and Ghosh, 2009). Studies have reported that patients with CLE fare worse than their counterparts with other dermatological conditions such as acne or nonmelanoma skin cancer (Klein et al., 2011, Vasquez et al., 2013) and experience a high burden of psychiatric morbidity, especially anxiety and depression (Achtman et al., 2016, Ferraz et al., 2006, Ishiguro et al., 2014).

Because patients’ perceptions can differ from clinicians’, it is important to use subjective measures, such as patient-reported outcomes (PROs), to capture quality of life (QoL) in patients (Ogunsanya et al., 2017). However, current QoL measures do not capture all issues relevant to CLE because patients with CLE were not well represented in the development of the current instruments (Chren et al., 1996, Finlay and Khan, 1994). For example, the Skindex (Chren et al., 1996), a commonly used QoL questionnaire for skin diseases, lacks questions that address concerns unique to patients with CLE such as avoidance of sun exposure. To improve assessment of therapeutic efficacy in CLE, a better understanding of the disease burden of CLE is required.

Little is known about the subjective experiences of patients with CLE. Such information would be vital for providers to optimize patient care, advise patients on appropriate coping strategies, and develop better PROs. Thus, focus groups (FGs), a well-known approach to elicit perspectives from patients (Rabiee, 2004), were used to explore patients’ views on how CLE has affected their lives and the unmet needs with regard to CLE treatment and care.

## Methods

Patients were recruited via the University of Texas (UT) Southwestern Cutaneous Lupus Registry. Patients from this registry were invited to participate in the study by phone, e-mail, or in person. Due to concerns with regard to transportation to the data collection site at the UT Southwestern Medical Center, only patients who lived in the Dallas-Fort Worth metroplex were contacted.

Inclusion criteria included patients who had a diagnosis of CLE, were age ≥ 18 years, and were able to understand written and spoken English. The institutional review boards at UT Austin (2015-09-0041) and UT Southwestern (STU 102015-056) approved this study. Participants received a $50 VISA gift card for their time.

### Data collection

FGs were utilized to a) better understand the impact of CLE on patients’ lives within the context of lived experiences (Rabiee, 2004) and b) build upon the limited knowledge of issues that are relevant to PRO measures in CLE (Wackerbarth et al., 2002). To this end, three FGs were conducted in February 2016. Two trained individuals experienced in qualitative interviews served as the moderator (M.E.O.) and assistant notetaker (D.L.).

FG discussions were audio-recorded and transcribed verbatim (M.E.O., C.M., and F.I.) for data analyses. After verbal consent had been obtained, patients completed a brief demographic survey (e.g., age, sex, years since diagnosis, race/ethnicity, and smoking status) for descriptive purposes. Patients then verbally answered questions about CLE’s impact on their QoL and unmet needs with regard to treatment and management (Table 1). FGs were conducted until the point of saturation was reached.

**Table 1:** Focus group discussion guide

Understanding the impact of CLE on patients’ lives
1. Briefly tell me/write down all the ways that CLE affects you. ^a^Probe: Kindly tell me how CLE affects your work life, daily activities, social life, personal relationships, leisure activities, or any other ways possible. Also tell me the impact of CLE on photosensitivity, alopecia, and your mental health.
2. Which other areas can you think of that has been affected by CLE?
Unmet needs with regard to CLE treatment and care
3. Thinking about your treatment for CLE, what types of issues are important to you? Why is that? aProbe: What attributes would you like to see in a future therapy for CLE that isn’t currently available in your current regimen?

CLE, cutaneous lupus erythematosus

a Probes were used as necessary and appropriate.

### Data analysis

Demographic responses were analyzed using descriptive statistics (e.g., frequencies, means, and standard deviations) on SPSS version 23. FG transcripts were reviewed and content-analyzed to identify emerging themes related to overall QoL using Braun and Clarke’s (2006) approach for content analysis. The first step in this process was familiarization with the data through repeated active readings of the entire transcript prior to identification of possible patterns and codes. Then initial codes were derived by working systemically through the entire data set to identify concepts that could be collapsed to form recurring patterns (themes). Validity checks were conducted throughout the data analysis by checking and rechecking the concepts. Using topic coding, recurring concepts and phrases were grouped into codes, and subthemes were placed under overarching themes/categories. The themes were refined, categorized, and renamed in a meaningful way. Qualitative data analysis was supported through the use of Dedoose Version 6.1.18, 2015.

To ensure accuracy and reliability of the identified themes, three coders (M.E.O., F.I., and C.M.) independently pretested the definitions of the themes/categories by thoroughly reviewing the transcripts. Results were compared among the three coders, and any coding discrepancies were resolved through discussion. The final themes, subthemes, and representative quotes were presented to the research team for review and group discussion. Using an interactive process, the final themes were tested against the data by checking with five of the FG participants. The role of each author along with the guidelines set by Braun and Clarke (2006) are outlined in Table 2.

**Table 2:** Braun and Clarke (2006) thematic analysis guidelines and role of authors

1. Familiarizing yourself with your data	The accuracy of the transcripts was checked. Transcripts were read by M.E.O., C.M., and F.I., and initial thoughts were written down and discussed as a team.
2. Extracting initial codes	After transcription, the FG data were de-identified into Dedoose (Dedoose Version 6.1.18, 2015), where the data were analyzed for themes All transcripts were coded systematically using inductive methods. The three interviews were coded by M.E.O., C.M., and F.I., and inconsistencies were resolved by rereviewing the transcripts until a consensus was reached. Using Dedoose, representative quotes were added to the initial codes extracted.
3. Generating themes	From the initial codes extracted by M.E.O., C.M., and F.I. and by thorough review of the transcripts, meaningful themes were created. A preliminary description of the main and subthemes was made.
4. Reviewing themes	To achieve this step, M.E.O. checked the preliminary description of themes with the original data (transcripts). Inconsistencies were discussed in the research group. The themes and subthemes were also rereviewed by M.E.O., C.M., and F.I.
5. Defining and naming themes	The thematic content was created by M.E.O. initially, who then worked with C.M. and F.I. to generate the storyline. The results generated from this step were presented to qualitative research experts and cutaneous lupus erythematosus health care providers to obtain feedback on refining the output.
6. Producing the report	The first draft of the report was written by M.E.O. who, together with F.I. and C.M., also selected representative quotations to illustrate the themes. B.F. and C.B. reviewed the draft and provided input on necessary adjustments to be made. Finally, all members of the research team reviewed the report for accuracy, and their responses were recorded.

## Results

A total of 19 patients participated in the FGs. The average age of was participants was 49 ± 14 years and average age at CLE diagnosis was 31 ± 10 years. Most participants were female (94.7%), African-American (68.4%), non-Hispanic/Latino (89.5%); had chronic CLE (73.7%); and had a concomitant diagnosis of systemic lupus erythematosus (SLE; 57.9%). Table 3 summarizes patients’ demographic and disease characteristics.

**Table 3:** Patient demographic and disease characteristics (N = 19)

Characteristic	Frequency (%)	Mean ± SD
Age (y)		49 ± 14
Age at diagnosis (y)		31 ± 10
Sex		
Female	18 (94.7)	
Male	1 (5.3)	
Race		
African-American	13 (68.4)	
Caucasian	6 (31.6)	
Ethnicity		
Not Hispanic/Latino	17 (89.5)	
Puerto Rican	1 (5.3)	
Mexican-American	1 (5.3)	
Geographic residence		
Urban	10 (52.6)	
Suburban	6 (31.6)	
Rural	3 (15.8)	
Marital status		
Single, not in a relationship	6 (31.6)	
Married	6 (31.6)	
Unmarried, in a relationship	4 (21.1)	
Divorced/separated	2 (10.5)	
Partner/living together	1 (5.3)	
Education		
College degree	9 (47.4)	
Less than high school/GED	7 (36.8)	
Postgraduate	3 (15.8)	
Health insurance		
Private insurance (e.g., BlueCross/Blue Shield)	9 (47.4)	
Public insurance (e.g., Medicaid, Medicare)	7 (36.8)	
None/self-pay	3 (15.8)	
Smoking status^a^		
Nonsmoker	12 (63.2)	
Current smoker	4 (21.1)	
Predominant CLE subtype		
Chronic	14 (73.7)	
Subacute	3 (15.8)	
Acute	2 (10.5)	
Concomitant diagnosis of SLE		
Yes	11(57.9)	
No	8 (42.1)	
Perception of health		
Fair	9 (47.4)	
Good	9 (47.4)	
Excellent	1 (5.3)	

CLE, cutaneous lupus erythematosus; GED, general equivalency diploma; SLE, systemic lupus erythematosus

a Three patients had missing data

Saturation was reached after the third FG. Four themes emerged and captured patients’ perceptions of how CLE impacts their lives. Themes, subthemes, and sample quotes are listed in Table 4 and summarized in the following sections.

**Table 4:** Major themes and subthemes by participants

Major themes	Subthemes	Sample quotations
**Disease sequelae of CLE**	Acute manifestations	- “My skin hurts and I mean physically hurts.” - “It itches, but you’re told not to scratch it—it’s best just to try and rub it, you know?” - “I get all these bumps, swelling, and stuff happening to me and I don’t know where it is coming from. I break out a lot, bumps everywhere.” - “People see my ear burnt and think it is dirty. I don’t explain it. It’s nothing and has not affected the way I breathe.
	Chronic manifestations	- “For me, I am not totally vain but I think that’s just the main thing for me. When I was younger, I had pretty really thick hair which I used to wear short and got a lot of compliments for. I also had a great hair stylist. When I started losing it to the point when it was scarring and it didn’t come back it got to a point, it’s different. I get tired of the weaves and the wigs. There’s no way I can just go natural even because I have so much scarring.” - “I’m self-conscious about is that ‘wolf mask’ that comes through here [nasal ridge over face]. Just the color of these and that mask stayed here. And I didn’t want to take pictures, facial pictures or anything of that nature.”
	Medication effects	- “I take methotrexate for my skin. I mean I don’t like it because it is a chemotherapy drug and it is hard on your liver, but I want quality of life. If my life is shortened because of all this stuff I have been taking since I was 19. I still take prednisone. I have had to accept it. I need to find a way to enjoy life.” - “That is why I don’t take my meds a lot. I feel like it be messing me up more than it is helping me. You know, once I read those side effects it is downhill for me because I am not about to take it. I am already bald.”
**Effects of CLE on social interaction**	Social anxiety	- “CLE makes me not wanting to get around people. I feel like being alone.” - “And so I’ve been told sometimes that I use lupus as a crutch or an excuse to behave a certain way. There are days when you just don’t want to interact. I know that I don’t know where the mood swings come from but it could be there.”
	Public misconceptions and education	- “I went to 7-Eleven and someone said, ‘What man did you like that?’ I jumped up and left home right quick. I didn’t think about putting my makeup on. I look at it like this: It’s some ignorant people in the world. And instead of staring at me like a child, ask me what’s wrong, ‘cause I have a bad mouth.” - “I just don’t know how to explain it to people. You know, I have a really good friend, she knows I have lupus and that is all she knows. She doesn’t know what it is. How do you explain it to people? Like I don’t just feel like it today?”
	Seriousness and unpredictable nature of the disease	- “I was planning to become a police officer and in the police academy in Dallas. In the NW division, I’d been in the Police Exploration program ever since I was 14 and that was a really big dream for me. Once I started having these symptoms and then I realized that I had lupus, you get this diagnosis and then you start getting sick all the time. You start losing your strength and endurance. You can’t keep up with the team anymore. You just feel like you can’t do what you used to do, like you are not capable of it. So, yes, it has changed my plans a lot. I went to school and I had to take semester-long breaks because I will just spend months at a time in the hospital.” - “Like she said, it is very depressing. One week you may have a good week and think you are cute and stuff then the next week comes and your face is all jacked up and stuff. It is an up and down thing.”
**CLE effects on functioning**	Mental effects	- “I spent a month at a mental hospital for a suicide attempt and that was my eighth attempt. I don’t remember... I don’t remember if I’d struggled with depression before I was diagnosed with lupus. - “I know that I don’t know where the mood swings come from but it could be there. I don’t know where the depression comes from but it could be there. It may not have anything to do with any of this be it systemically or the outer. It’s just what it is.”
	Body image	- When I was first put on prednisone, I was on 60 mg and went up to 160 lbs. For me, that was really, really bad because I was a soccer player. I weighed 104 lbs and that was my normal weight.” - “I am real self-conscious about my skin. I would want my skin not to break out a lot. When it happens, I can’t stand it and so I would want that to go away real fast.”
	Coping mechanisms	- “Spending time with my kids. My kids make me want to be around because I want to live for them.” - “I stay high [on recreational drugs]. I am not going to lie. I do.” - “I don’t want to think about it [lupus]. I just stay busy.”
**Unmet needs**	Symptom-related	- “Something to reduce scarring, the damage caused by the disease. I have it a lot on my back and stomach. I have scars everywhere on my body and back. I know there’s stuff out there, but it is expensive.” - The discoloration, can we all just clear them altogether so no one has to go through this, okay?” - “It (hair) should come back. A success for me will be measured by my hair growing back.”
	Treatment-related	- “I would have to see a reduced number of flares and an improvement in my lab work. This is important to me because I spend way too much time in the hospital. A 60% reduction in hospitalization would be a success for me.” - “I take over 23 medications a day, uh uh, too many!” - “I think something for mental and emotional balance is very important because I know that that has helped me.”
	Support system	- “There are no group of people that can help you with how you can blend and cover scars, or what to use to cover my hair. There’s no one, you have to go out there to figure out to so much by yourself. There’s no support.” - “They have that for cancer patients. My godmother is a breast cancer survivor, and I went to a seminar. They gave them this little bag that had all these makeup and kit.”

CLE, cutaneous lupus erythematosus

### Theme 1: Disease sequelae of cutaneous lupus erythematosus

#### Acute manifestations

Multiple acute cutaneous manifestations included pain, itching, swelling, burning, and photosensitivity. These acute manifestations occurred sporadically and would often prevent patients from engaging in outdoor activities due to fear of exacerbating symptoms.

“I used to play soccer and I loved being outside, especially in the summertime. Now, I can’t even be outside for a long time without having to feel like my face is burning.”

“Three weeks ago, my mouth was swollen and there were bumps everywhere on my face. It was horrible, okay? I wanted to die. It was just that horrible, so I went to the doctor.”

Itching was problematic when not controlled by medication. One patient noted:

“Everything itches on me; I take all of my clothes off and take a shower.”

Itching also appears to increase due to active lesions, as typified below:

“I think that in particular, I used to get a pimple and a little red thing like an eruption on my scalp. My scalp also doesn’t itch as much as it used to. With treatment, it has helped me a lot.”

#### Chronic manifestations

Scarring, hair loss, and dyspigmentation were examples of chronic manifestations of CLE. Thus, patients resorted to the use of cover-ups such as wigs (for female patients), long jackets, hats, or sunglasses to conceal the physical signs of the disease. Furthermore, most patients reported summertime as the most difficult season of the year because they could no longer wear bathing suits or engage in outdoor activities due to photosensitivity, visible discoloration, and scarring.

“I can’t wear a bathing suit either because of all the scars that I have. I just don’t feel comfortable, and I have scars all over my chest as well. It also impacted my outside life.”

“I would wear these crazy long wigs and, you know when it was brand new, and cover my face in this makeup that didn’t match. They were like orange, you know, and ridiculous.”

The permanent and visible skin lesions such as scarring and dyspigmentation also often led to embarrassment. Patients were often more distressed by what others thought of their physical appearance than by their signs and symptoms.

#### Medication effects

Patients raised several concerns regarding the toxicities and number of medications they were taking. One female patient expressed regret for her inability to have more children because of the teratogenic effects of thalidomide. Most patients reported taking an excessive number of pills to manage their disease. In addition, some patients feared that more medications could be added as their disease progressed. Because of the limited treatment options, patients expressed the need for more drugs to be developed for CLE. One patient displayed her frustration by saying:

“It’s like since I was diagnosed 30 years ago and there has been no difference in the treatment. I am still using the same medications as I did many years ago. I wished there were different treatment options.”

### Theme 2: Effects of cutaneous lupus erythematosus on social interaction

#### Social anxiety

Patients not only found it hard to interact with others but were often worried that they would be a burden to others. This affected their relationships with other people, especially romantically. One patient noted:

“I was married one time and it kind of messed up my relationship with my husband because he felt like I was always tired or I will always complain about my face or something.”

Other patients also reported that their friendship circle had thinned out:

“I had to separate myself from my friends; at first, they kinda judge you. They are like, ‘Oh, you are sick again.' What do you mean sick again? I have been sick. They don’t really understand what you are going through.”

#### Public misconceptions and education

Patients also indicated that they had been labeled as illicit drug users (due to excessive use of cover-up clothing), victims of domestic violence (due to visible skin discoloration), or contagious (due to active lesions). One patient explained:

“I used to be a manager, and I have rashes on my hand. People will look at me funny like they were disgusted to take money from me.”

Patients expressed the need for a proper way to educate the public about CLE. Particularly, patients yearned for more compassion and understanding from the public and hoped that CLE would receive attention similar to that paid to other diseases such as HIV infection and cancer.

#### Seriousness and unpredictable nature of cutaneous lupus erythematosus

Most patients, especially younger ones, reported being unable to make long-term career goals due to the uncertainty of the timing and frequency of disease flares. Consequently, they had to give up their jobs, which led to increased worry and anxiety. Even on a daily basis, patients reported how sudden flares would render them unable to perform menial tasks, thus increasing their dependence on others for assistance. One patient commented:

“I think it is very difficult for people to understand because of the ups and downs. A lot of times you are feeling good, and they don’t understand the quick changes. Again, I am a mother. With my kids, a lot of times I don’t feel like doing anything with them because of my lack of energy. It is very hard for me.”

### Theme 3: Cutaneous lupus erythematosus effects on functioning

#### Mental effects

Self-reported depression, insomnia, and anxiety were core concerns of patients with CLE. Patients who reported being depressed also talked about having suicidal attempts (or thoughts), mood swings, and avoidance of others. For example, one patient said:

“My confidence was so worn down that I stopped going out with friends because I might be somewhere and all of a sudden I don’t feel well. I have learned to navigate that through the years, but it has definitely affected me socially.”

Many patients reported severe sleep disturbance that ranged from problems in initiating sleep to maintaining a restful sleep. Whether these issues were related to medical side effects or anxiety as sequelae from their CLE is uncertain. Also, female patients with children, regularly worried about the possibility of passing CLE down to their children. In the words of one patient:

"My baby is 25, and I worry about that every day. My son, he doesn’t have it yet, but my brother does.”

#### Body image

Most patients expressed discontent with their body image due to visible skin changes, including scarring, alopecia, and dyspigmentation, and weight gain (from the use of steroids). Overall, patients expressed a desire to have the body they had prior to the onset of lupus symptoms.

“…that is why I probably ain’t gon’ have anybody because I can’t let nobody like me because I don’t like myself. It is hard to let someone in if you don’t like yourself. I don’t like it. I wanted to show everybody this [shows an old photo of self to the group]. This is me; I want to look like this again. I miss this person. I don’t like this person; I really don’t. That is what I am struggling with... not liking myself. I don’t like my face like this, I really don’t.”

#### Coping mechanisms

Most patients reported using positive coping strategies, which included relying on family members, friends, and religious faiths to help them through tough times. One patient explained:

“My faith helps me cope – praying and just reading the Bible.”

Several patients had developed resilience to the disease and found their “new normal.” Also, older patients appeared to have a better understanding of the disease course due to their greater experience at handling different challenges. On the other hand, patients also engaged in negative coping strategies to distract themselves from the harsh realities of having CLE. Examples included using recreational drugs, as summarized by the following quote:

“I stay high (on recreational drugs). I am not going to lie. I do.”

### Theme 4: Unmet needs

Several subthemes—symptoms, treatment-related, or social—were generated when patients were asked about their perceptions of unmet needs with regard to their treatment and care. For the symptoms and treatment-related needs, patients strongly expressed a desire for disease-modifying therapies that can mitigate the signs and symptoms associated with both acute (e.g., itching, burning) and chronic (e.g., scarring, discoloration, alopecia) disease or reduce the number of pills they take. For social needs, patients recommended a starter kit to navigate coping with CLE, especially when newly diagnosed. Many patients talked about how it was often difficult to figure out practical steps to deal with CLE, such as grooming tips (e.g., cover-up makeup that matches skin color) to conceal visible skin lesions. One patient referred to breast cancer as an example:

"They have that for cancer patients. My godmother is a breast cancer survivor, and I went to a seminar. They gave them this little bag that had all these makeup kits.”

## Discussion

This study sought to characterize individual patients’ perspectives of living with CLE and to elicit important issues with regard to their treatment and care through FG interviews. While confirming CLE’s negative impact on QoL (Foering et al., 2012, Ishiguro et al., 2014, Klein et al., 2011, Vasquez et al., 2013), our study’s findings delineate the disease sequelae, the impact on social interaction and functioning, and patients’ unmet needs. Furthermore, we will use this study to help develop a CLE-specific QoL measure that we postulate will improve assessments of the impact of CLE disease burden. Initial efforts to create a CLE-specific QoL measure began with the addition of three items to the Skindex-29 to assess photosensitivity and alopecia (Foering et al., 2012, Vasquez et al., 2013). We hypothesized that additional CLE-relevant issues other than photosensitivity and alopecia could be included in a revised CLE-specific QoL measure, so we conducted this qualitative study in patients with CLE to identify other disease-relevant concerns.

Our FGs elicited negative sentiments on permanent alterations in physical appearance, including scarring and dyspigmentation. Patients employed grooming practices such as wearing long-sleeved clothing, heavy makeup, and wigs to cover their chronic skin lesions. Current QoL instruments used for CLE clinical trials such as the Skindex and Dermatology Life Quality Index (Ky et al., 2015, Okon et al., 2014, Szepietowski et al., 2013) do not include questions on these chronic features of CLE, such as scarring and dyspigmentation. A study by Verma et al. (2014) reported no significant correlation between QoL and dyspigmentation and scarring.

One explanation is that the QoL instrument used may not have had enough questions related to exploring disease damage and their chronic representations in patients with CLE. Adding questions that probe the extent to which patients cover their CLE lesions and alter their grooming practices in a revised CLE-specific QoL questionnaire would help address this deficiency. In addition, because the chronic sequelae of CLE can affect one’s well-being, clinicians may serve as sources for referral to specialists for psychiatric evaluations. Psychiatric therapy, such as cognitive behavior therapy, has been shown to be effective in patients with dyspigmentation and scarring (Papadopoulos et al., 1999, Van Loey and Van Son, 2003).

Like SLE (McElhone et al., 2010), CLE confers social difficulties on patients. Due to the unpredictability of the disease, patients find it hard to sometimes foster interactions with those around them. By retreating and isolating themselves, patients may be at a greater risk of worsening their mental health. Providers can seek out behaviors that are suggestive of social isolation by asking patients about their preference to stay at home or do things alone due to their CLE. Several patients expressed frustrations about how CLE is often perceived by others as less severe because it is skin-related with mostly subjective signs and symptoms. Educating the public about CLE would help heighten understanding of this disease, minimize stigmas, and provide support and empathy to patients with CLE.

Body image issues, which were often due to chronic manifestations of CLE, were strongly expressed throughout the FG themes. Concerns with body image have been linked to depression and other mental-related illnesses (Cornwell and Schmitt, 1990, Jolly et al., 2012, Monaghan et al., 2007). In studies that involve patients with SLE, body image issues were more commonly associated with the negative effects from treatments than with the disease itself (Cornwell and Schmitt, 1990, Jolly et al., 2012, Monaghan et al., 2007). Because body image has yet to be extensively examined in patients with CLE, future QoL studies can incorporate questions on body image to capture disease effect and treatment impact on patients.

Positive and negative coping strategies were cited by several patients with CLE. Patients (especially newly-diagnosed ones) who receive psychosocial support from friends and family have fared better with their disease management and have participated more in their healthcare (Gustafson et al. 2001). Furthermore, anecdotal comments made by FG participants showed that patients valued being with other patients with CLE in a safe environment where they can share their concerns, needs, and experiences. Physicians and caregivers who treat patients with CLE can link these patients with lupus patient support groups that can support positive mental health. On the contrary, some patients noted maladaptive coping strategies such as illicit drug use and social isolation. Studies in patients with SLE have reported strong correlations between the use of maladaptive (negative) coping strategies and depression, physical disability, and social disability (Kozora et al. 2005). Providers can also ask patients about their coping strategies so that negative strategies can be addressed with appropriate interventions.

Patients with CLE also strongly expressed a desire for medications to improve acute symptoms and chronic skin sequelae. Improving chronic manifestations is vital; several patients expressed frustrations with the constant use of cover-ups to mask such visible CLE manifestations. Further, clinical trials in patients with lupus often use improvement in Cutaneous Lupus Activity and Severity Index activity scores but do not report changes in damage scores that measure the amount of scarring and dyspigmentation (Khamashta et al., 2016, Okon et al., 2014). Although there are no known medications shown to improve chronic manifestations of CLE, our study raises the importance of developing medications that alleviate both acute and chronic skin sequelae, especially those that would treat patients as a whole; these would likely be viewed as a therapeutic success by patients.

This study has several limitations. The small sample size may not be representative of the larger population of patients with CLE. Even though data saturation was reached at the end of the third FG, our data may not have captured the gamut of issues that are important to patients with CLE. Finally, participants were predominantly female with only one male participant, which may have enhanced gender-specific issues. Nonetheless, CLE is a female-predominant disease with at least a 4:1 ratio of female to male patients (Klein et al., 2011, Vasquez et al., 2013).

## Conclusions

This study outlines several themes that captured the burden of CLE on patients; these ranged from disease sequelae and social effects to functioning. As a result, patients used several positive and negative coping mechanisms to mitigate the effects of CLE. Providers can look for potential warning signs in patients by screening for mental health issues and checking for negative coping strategies. We propose improving our assessment of patients’ perspective of disease skin burden by expanding existing QoL instruments such as the Skindex to incorporate other CLE-specific attributes such as body image, scarring, and dyspigmentation. This will augment patients’ voices in determining which medications for CLE reach therapeutic success.
